# Exposure to Fine Particulate Matter during Pregnancy and Risk of Preterm Birth among Women in New Jersey, Ohio, and Pennsylvania, 2000–2005

**DOI:** 10.1289/ehp.1307456

**Published:** 2014-05-30

**Authors:** Kristen M. Rappazzo, Julie L. Daniels, Lynne C. Messer, Charles Poole, Danelle T. Lobdell

**Affiliations:** 1 Department of Epidemiology, UNC Gillings School of Global Public Health, University of North Carolina at Chapel Hill, Chapel Hill, North Carolina, USA; 2 School of Community Health–College of Urban and Public Affairs, Portland State University, Portland, Oregon, USA; 3 U.S. Environmental Protection Agency, Office of Research and Development, National Health and Environmental Effects Research Laboratory, Chapel Hill, North Carolina, USA

## Abstract

Background: Particulate matter ≤ 2.5 μm in aerodynamic diameter (PM_2.5_) has been variably associated with preterm birth (PTB).

Objective: We classified PTB into four categories (20–27, 28–31, 32–34, and 35–36 weeks completed gestation) and estimated risk differences (RDs) for each category in association with a 1-μg/m^3^ increase in PM_2.5_ exposure during each week of gestation.

Methods: We assembled a cohort of singleton pregnancies that completed ≥ 20 weeks of gestation during 2000–2005 using live birth certificate data from three states (Pennsylvania, Ohio, and New Jersey) (*n* = 1,940,213; 8% PTB). We estimated mean PM_2.5_ exposures for each week of gestation from monitor-corrected Community Multi-Scale Air Quality modeling data. RDs were estimated using modified Poisson linear regression and adjusted for maternal race/ethnicity, marital status, education, age, and ozone.

Results: RD estimates varied by exposure window and outcome period. Average PM_2.5_ exposure during the fourth week of gestation was positively associated with all PTB outcomes, although magnitude varied by PTB category [e.g., for a 1-μg/m^3^ increase, RD = 11.8 (95% CI: –6, 29.2); RD = 46 (95% CI: 23.2, 68.9); RD = 61.1 (95% CI: 22.6, 99.7); and RD = 28.5 (95% CI: –39, 95.7) for preterm births during 20–27, 28–31, 32–34, and 35–36 weeks, respectively]. Exposures during the week of birth and the 2 weeks before birth also were positively associated with all PTB categories.

Conclusions: Exposures beginning around the time of implantation and near birth appeared to be more strongly associated with PTB than exposures during other time periods. Because particulate matter exposure is ubiquitous, evidence of effects of PM_2.5_ exposure on PTB, even if small in magnitude, is cause for concern.

Citation: Rappazzo KM, Daniels JL, Messer LC, Poole C, Lobdell DT. 2014. Exposure to fine particulate matter during pregnancy and risk of preterm birth among women in New Jersey, Ohio, and Pennsylvania, 2000–2005. Environ Health Perspect 122:992–997; http://dx.doi.org/10.1289/ehp.1307456

## Introduction

Particulate matter (PM) ≤ 2.5 μm in aerodynamic diameter (PM_2.5_), one of the criteria air pollutants regulated under the Clean Air Act (2012), is a complex mixture of extremely small particles and liquid droplets. PM_2.5_ may be a carrier for hazardous compounds such as polycyclic aromatic hydrocarbons and metals, which particulates absorb. Although levels of PM_2.5_ vary across the United States, and are often below U.S. Environmental Protection Agency (EPA) standards [24-hr standard, 35 μg/m^3^ (U.S. EPA 2012, 2013b)], everyone is exposed to some extent. PM_2.5_ has been associated with adverse health outcomes, including cardiovascular mortality, lung cancer, asthma, and adverse pregnancy and birth outcomes (Backes et al. 2013; Dominici et al. 2003, 2006; Lewtas 2007; U.S. EPA 2009). Of the birth outcomes studied in conjunction with PM exposure, preterm birth (PTB) is an important marker for fetal underdevelopment, conveying risk for further adverse outcomes, including infant mortality and problems with neurodevelopment and growth (Behrman and Butler 2007; Gilbert et al. 2003; Mathews and MacDorman 2010; Saigal and Doyle 2008). Many studies have reported that PTB is positively associated with PM_2.5_ over whole pregnancy, first trimester, and late pregnancy exposures (Brauer et al. 2008; Chang et al. 2012; Gehring et al. 2011; Huynh et al. 2006; Lee et al. 2012; Warren et al. 2012; Wilhelm et al. 2011; Wu et al. 2009, 2011), although others have reported null or inverse associations of PM_2.5_ on PTB (Darrow et al. 2009; Gehring et al. 2011; Jalaludin et al. 2007; Wilhelm and Ritz 2005). Meta-analyses have found overall increases in associations between PM_2.5_ and PTB [e.g., Sapkota et al. (2012), with PM_2.5_ exposure in the third trimester odds ratio (OR) = 1.07 (95% CI: 1.00, 1.15)], but noted that variable results across studies may be attributable to differences in study designs, populations, or exposure metrics and contrasts (Sapkota et al. 2012; Stieb et al. 2012). Most studies have relied on air monitoring for exposure assignment, limiting inclusion to women residing close to active monitors during pregnancy. Additionally, reliance on a few central monitors assumes no spatial variation in ambient PM_2.5_ concentrations, which may result in exposure misclassification. Most also examine exposure windows spanning a month or a trimester in length, which may mask temporal variability. Finally, previous studies have focused on any births between 20 and 36 weeks, yet etiology of PTB may vary over this period.

## Objectives

In this study, we examined the association between ambient PM_2.5_ and risk of PTB using a cohort of singleton pregnancies that had completed at least 20 weeks of gestation during 2000–2005 across three states (Pennsylvania, Ohio, and New Jersey). We employed output from the U.S. EPA’s Community Multiscale Air Quality (CMAQ) model (Hogrefe et al. 2009), which offers complete spatial coverage and daily estimated air pollutant concentrations, leading to an extensive study area and population. We classified preterm births into four categories (20–27, 28–31, 32–34, and 35–36 weeks completed gestation) and estimated risk differences (RDs) for each category in association with a 1-μg/m^3^ increase in PM_2.5_ exposure during each week of gestation.

## Methods

*Study population*. We used live birth records provided by the State Health Departments of Pennsylvania, New Jersey, and Ohio to construct a cohort of those at-risk for PTB (i.e., achieving a gestational age of at least 20 weeks) between 1 January 2000 and 31 December 2005. These states have similar source profiles for PM_2.5_ (e.g., electricity generation is the main source of PM_2.5_ for Ohio and Pennsylvania), or PM is from regional sources (e.g., air pollutants are transported from the Ohio River valley area to Pennsylvania and New Jersey), so PM composition should be similar in these areas (Garcia et al. 2011; U.S. EPA 2013a). From all birth records (*n* = 2,495,350), the study population was restricted to singleton pregnancies with no recorded birth defects at time of birth, with an estimated gestational age available, and having achieved gestational week 20 no earlier than 1 January 2000 and gestational week 44 no later than 31 December 2005 (birth data set, *n* = 2,142,915/excluded *n* = 352,435). Gestational age requirements are necessary so that each pregnancy would have been entirely observable within the study period no matter at which point birth occurred, and to avoid fixed-cohort bias where the beginning of the study misses shorter pregnancies and the end misses longer pregnancies (Barnett 2011; Strand et al. 2011). A point-geocodeable (latitude and longitude assignable) birth address was also required (excluded *n* = 202,702). After restrictions, the final study population included 1,940,213 pregnancies.

*Gestational age, pregnancy start, and preterm birth status*. Gestational age was determined by clinical estimate of gestational age as reported on birth certificates. Estimated date of last menstrual period (LMP) was calculated by subtracting clinical estimate of completed gestational weeks from date of birth. PTB status was defined as having a gestational age between 20–36 completed weeks. PTB was subset into four categories based on definitions from the World Health Organization (2012): extremely PTB (ExPTB), gestational age 20–27 weeks; very PTB (VPTB), gestational age 28–31 weeks; moderate PTB (MPTB), gestational age 32–34 weeks; and late PTB (LPTB), gestational age 35–36 weeks. Term births were between 37 and 44 completed gestational weeks.

*Exposure data*. Maternal address data were taken from birth records and processed with the Zp4 address locator program (Semaphore Corporation, Monterey, CA) to assign latitude and longitude values (*n* = 2,042,425). Addresses that were not assigned latitude and longitude values (*n* = 452,925) were geocoded using the ArcGIS online geocoding service in ArcMap10 (ESRI, Redlands, CA), which returned 197,125 matched addresses and 8,949 addresses that were equally matched to two or more points and were then hand matched to the best candidate. In total, 2,248,499 pregnancies had latitude and longitude values. After restriction to pregnancies meeting all inclusion criteria, the study population was 1,940,213.

Daily estimated concentrations of PM_2.5_ were provided by the U.S. EPA’s Atmospheric Exposure Integration Branch for 1999 to 2005 in 12-km grids (Hogrefe et al. 2009). These estimates were constructed using output from CMAQ, bias-corrected with monitoring network data, as detailed in Hogrefe et al. (2009). Briefly, meteorological conditions and criteria pollutant emissions are input into CMAQ, which simulates atmospheric processes and estimates gridded concentrations of ambient air pollutants (Byun and Schere 2006; Hogrefe et al. 2009). Grids were matched to monitoring sites and a filter applied to created baseline concentrations of PM_2.5_. Adjustment factors were then created as the ratio of observed to modeled concentrations, spatially interpolated across the gridded field, and multiplied by CMAQ output to produce the final bias-corrected concentration estimates (Hogrefe et al. 2009). Both spatial and temporal variability in PM_2.5_ concentrations are present in our study.

We assigned daily values for pollutant exposure to pregnancies by matching geocoded maternal residential location to CMAQ grid. Each day from the calculated start of pregnancy to birth was matched to date of CMAQ concentration estimation. Exposure was assigned in two ways. First, we assigned average weekly exposures beginning on the estimated LMP date, such that week 1 comprised the estimated LMP date and the following 6 days. Second, we lagged weekly exposures backwards from the date of birth, such that lag 0 represented average PM_2.5_ during the week of birth (including the day of birth and the 6 days before birth), lag 1 represented average PM_2.5_ during the previous week (i.e., 7–13 days before birth), up to lag 8.

*Confounders and effect measure modifiers*. To achieve a least biased estimate of association, we identified potential confounders through directed acyclic graph (DAG) analysis. We constructed the DAG based on review of previous literature and knowledge of factors influencing PTB and air pollution. From the birth certificate, we included maternal race/ethnicity, education level, marital status, age at delivery, maternal smoking status, prenatal care initiation, and parity; and from the CMAQ model we included daily average ozone. We also considered unmeasured factors, such as infection status of mother during pregnancy, and general/area-level environmental quality. We used the DAG Program (Knüppel and Stang 2010) to identify minimally sufficient adjustment sets. Identified covariates included maternal race/ethnicity, education level, marital status, age at delivery, and ozone. Maternal demographic factors are risk factors of PTB and associated with socioeconomic status, which can influence where a woman resides and therefore PM_2.5_ exposure. Ozone has been associated with PTB and co-occurs temporally and spatially with PM_2.5_ (Lee et al. 2012; Olsson et al. 2013). We assessed effect measure modification (EMM) to reveal potential differences in the association. We also assessed potential modification by state, region, and population density, as proxy indicators of variation in PM_2.5_ composition.

*Statistical analysis.* Adjusted RDs were estimated using modified Poisson regression with an identity link; Poisson models produce equally valid estimates as binomial models, and though less efficient they are less likely to result in nonconvergence (Spiegelman and Hertzmark 2005; Zou 2004). We estimated absolute effect measures because they, unlike incidence ORs and other relative effect measures, are informative for public health impact and decision making, along with outcome severity (e.g., ExPTB, despite its relatively low frequency, is a much more adverse outcome than LPTB). We modeled each category of PTB separately as a dichotomous outcome. We included those at risk of PTB at a given time point in the model as appropriate (e.g., VPTB births were included in ExPTB models as non-events; those who had already experienced ExPTB were not included in VPTB models). PM_2.5_ was treated as a continuous variable. Individual models were produced for exposure during each week of gestation anchored from estimated LMP and each week lagged from birth. Models were adjusted for demographic characteristics (maternal age coded as restricted quadratic spline, others as in Table 1) and co-occurring ozone (continuous). To evaluate EMM, we ran separate models with an interaction term for each potential modifier and continuous PM_2.5_; interaction terms with *p* < 0.05 (due to the large population) were considered evidence of EMM. Potential modifiers were maternal race/ethnicity [black non-Hispanic, non-black (includes white non-Hispanic, Hispanic, and other)], smoking status (smoker, nonsmoker), parity (primiparous, multiparous), and infant sex. We performed sensitivity analyses to include temperature and season of conception as covariates. Because of concerns about residual confounding, we also examined smoking as a covariate. In addition, for comparability with previous work, we examined exposures averaged by trimester and entire pregnancy period. All analyses were performed using SAS version 9.3 (SAS Institute Inc., Cary, NC).

**Table 1:** Maternal and fetal characteristics according to preterm birth category for included pregnancies among women living in Ohio, Pennsylvania, or New Jersey (2000–2005) [*n* (%)].

Characteristic	ExPTB	VPTB	MPTB	LPTB	Term births
Observations (*n*)	8,664	12,004	31,446	90,037	1,639,376
Maternal education
Graduate school	550 (6)	933 (8)	2,865 (9)	9,245 (10)	202,783 (12)
Bachelor’s degree	1,021 (12)	1,651 (14)	4,688 (15)	14,964 (17)	325,596 (20)
Some college	1,905 (22)	2,604 (22)	6,982 (22)	20,429 (23)	372,682 (23)
High school diploma	3,221 (37)	4,227 (35)	10,789 (34)	29,566 (33)	491,888 (30)
Some high school	1,664 (19)	2,199 (18)	5,048 (16)	12,918 (14)	185,703 (11)
< 8th grade	303 (3)	390 (3)	1,074 (3)	2,918 (3)	60,724 (4)
Maternal race/ethnicity
Non-Hispanic white	4,120 (48)	6,549 (55)	18,848 (60)	58,868 (65)	1,152,731 (70)
Non-Hispanic black	3,279 (38)	3,671 (31)	7,781 (25)	17,034 (19)	225,430 (14)
Hispanic	1,009 (12)	1,337 (11)	3,435 (11)	10,004 (11)	177,708 (11)
Other	256 (3)	447 (4)	1,382 (4)	4,131 (5)	83,507 (5)
Maternal age
< 15	126 (1)	141 (1)	290 (1)	637 (1)	8,033 (< 1)
15–19	1,228 (14)	1,408 (12)	3,259 (10)	8,496 (9)	131,159 (8)
20–24	2,114 (24)	2,798 (23)	7,166 (23)	20,201 (22)	352,319 (21)
25–29	2,031 (23)	2,768 (23)	7,582 (24)	23,058 (26)	438,679 (27)
30–34	1,844 (21)	2,821 (24)	7,584 (24)	22,587 (25)	446,350 (27)
35–39	1,044 (12)	1,620 (13)	4,387 (14)	12,070 (13)	218,275 (13)
40–44	259 (3)	423 (4)	1,108 (4)	2,816 (3)	42,739 (3)
≥ 45	18 (< 1)	25 (< 1)	70 (< 1)	172 (< 1)	1,822 (< 1)
Abbreviations: ExPTB, extremely preterm births (20–27 weeks completed gestation); LPTB, late preterm births (35–36 weeks); MPTB, moderate preterm births (32–34 weeks); Term, term births (37–44 weeks); VPTB, very preterm births (28–31 weeks).					

This research was approved by the University of North Carolina at Chapel Hill’s Office of Human Research Ethics, the Pennsylvania Department of Health Bureau of Health Statistics and Research, New Jersey Department of Health and Senior Services Institutional Review Board, and the Ohio Department of Health Human Subjects Institutional Review Board. Informed consent was not required for this study because it was a secondary data analysis of existing data, and no participant contact was attempted.

## Results

Adjusted analyses included 1,781,527 of the 1,940,212 eligible pregnancies (not missing major covariates). Eight percent were preterm. Demographic profiles of women shifted with decreasing gestational age (Table 1; also see Supplemental Material, Table S1, for additional characteristics). Women with preterm pregnancies were less likely to have a bachelor’s degree or be married at time of delivery, and more likely to be non-Hispanic black than women with term pregnancies. The study population was primarily urban, with about 90% falling within metropolitan areas (data not shown).

Women who were excluded because they lacked residence location (and therefore exposure) data were more likely to be white, unmarried, less educated, younger, and to have a PTB (~ 9% vs. 8% in analytic population) than births to included women (data not shown).

Average weekly PM_2.5_ concentrations were similar across categories of PTB for exposure windows anchored at estimated LMP and lagged from birth, with means (± SDs) about 14.5 ± 5 μg/m^3^ and interquartile ranges around 6.2 μg/m^3^ (Table 2). Weekly PM_2.5_ concentrations were temporally correlated, with correlation coefficients for adjacent weeks of approximately 0.4, but dropping to near 0 for weeks further apart (data not shown).

**Table 2:** Descriptive statistics for PM_2.5_ (μg/m^3^) exposure concentrations, averaged over all weeks of exposure.

Statistic	ExPTB	VPTB	MPTB	LPTB	Term births
Minimum	3.73	3.55	3.15	2.84	2.45
25th	11.00	10.93	10.87	10.76	10.74
50th	13.8	13.72	13.65	13.54	13.51
75th	17.33	17.24	17.17	17.04	16.98
Maximum	50.82	50.87	53.33	55.19	58.25
Mean	14.62	14.54	14.47	14.36	14.31
SD	5.07	5.04	5.03	5.01	4.98
IQR	6.33	6.31	6.30	6.28	6.24
Abbreviations: ExPTB, extremely preterm births (20–27 weeks completed gestation); IQR, interquartile range; LPTB, late preterm births (35–36 weeks); MPTB, moderate preterm births (32–34 weeks); VPTB, very preterm births (28–31 weeks).					

**Figure 1:**
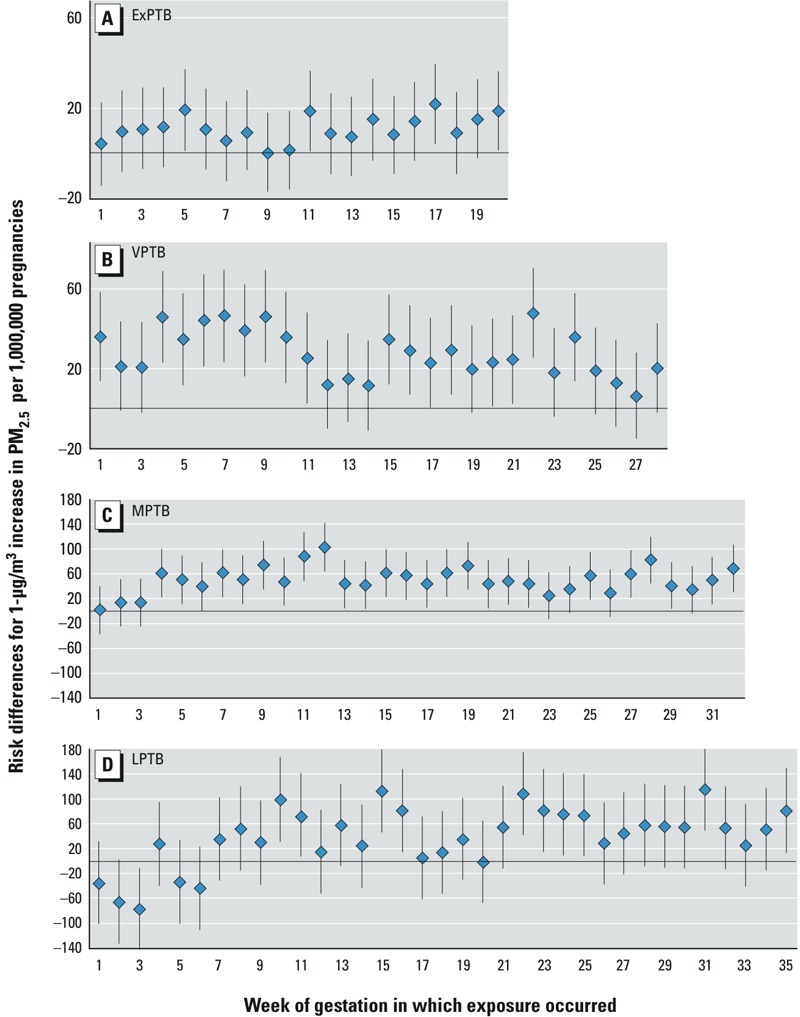
Results for PM_2.5_, exposures anchored at estimated LMP. Risk differences (95% CIs) for preterm birth with 1‑μg/m^3^ increases in PM_2.5_ per 1,000,000 pregnancies with exposures anchored at estimated LMP, for women residing in Ohio, Pennsylvania, or New Jersey with pregnancies at risk of preterm birth from 1 January 2000 to 31 December 2005. Risk of birth at (*A*) 20–27 weeks of gestation (ExPTB), (*B*) 28–31 weeks of gestation (VPTB), (*C*) 32–34 weeks of gestation (MPTB), and (*D*) 35–36 weeks of gestation (LPTB). Adjusted for maternal race/ethnicity, education level, marital status, age at delivery, and co-occurring ozone. Numeric estimates are provided in Supplemental Material, Table S2.

Results for exposure anchored at estimated LMP are presented in Figure 1. Scales vary between outcomes due to differing effect estimate and confidence interval magnitudes (numeric data are provided in Supplemental Material, Table S2). For ExPTB (Figure 1A), elevated RDs were observed with exposure to PM_2.5_ in gestational weeks 2–8 and weeks 11–20, with more consistency in early pregnancy. For VPTB (Figure 1B), elevations in RDs are highest with exposures at gestational weeks 4–9 and 15–24, though RDs are generally elevated for exposure in most weeks. For MPTB (Figure 1C), RDs were elevated with exposure to PM_2.5_ at gestational week 4 and increased with exposure through gestational week 12. RDs dropped at week 13, though remained positive through the rest of pregnancy. For LPTB (Figure 1D), associations were negative with the earliest weeks of exposure, with some positive though fluctuating RDs through week 20. However, after week 20, PM_2.5_-associated RDs were positive and remained elevated through week 35. Results with and without adjustment for ozone were similar (see Supplemental Material, Figure S1 and Table S3). Some commonalities are present between birth categories, such as a rising of associations in the first few weeks of pregnancy for VPTB and MPTP; however, there were also differences in patterns of associations with PM_2.5_ exposure across outcome categories, in particular for the earliest and latest PTBs.

**Figure 2:**
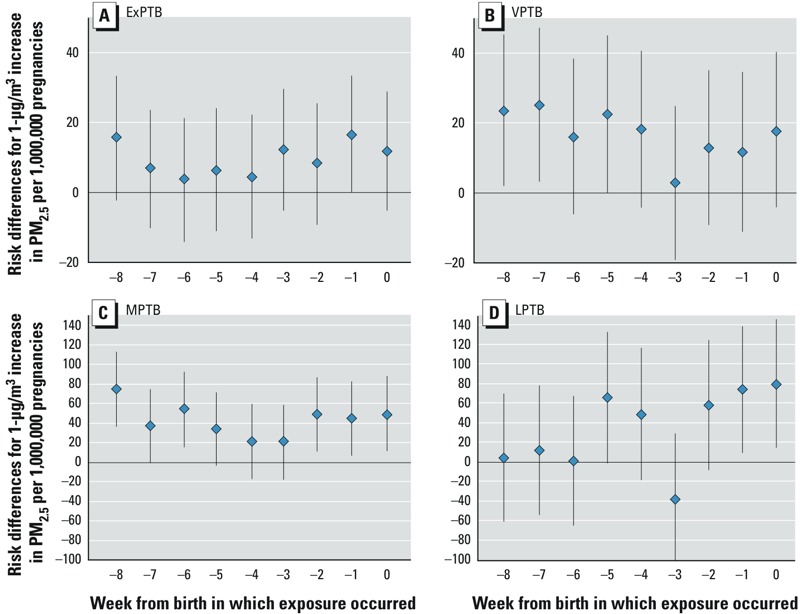
Results for PM_2.5_, lagged exposures. Risk differences (95% CIs) for preterm birth with 1‑μg/m^3^ increases in PM_2.5_ per 1,000,000 pregnancies with exposures lagged from birth, for women residing in Ohio, Pennsylvania, or New Jersey with pregnancies at risk of preterm birth from 1 January 2000 to 31 December 2005. Risk of birth at (*A*) 20–27 weeks of gestation (ExPTB), (*B*) 28–31 weeks of gestation (VPTB), (*C*) 32–34 weeks of gestation (MPTB), and (*D*) 35–36 weeks of gestation (LPTB). Adjusted for maternal race/ethnicity, education level, marital status, age at delivery, and co-occurring ozone. Numeric estimates are provided in Supplemental Material, Table S4.

Results for exposures lagged from birth are presented in Figure 2 (numeric data are provided in Supplemental Material, Table S4). RDs were consistently elevated for exposures 0–2 weeks before birth across PTB categories; but for exposure lagged further from birth, patterns across PTB categories were not consistent. RDs generally dropped to null around lag period 3, and then increased again for ExPTB, VPTB, and MPTB. LPTB RDs are positive for exposures with lags 0–3 and lags 4–5. Results with and without adjustment for ozone were similar (see Supplemental Material, Figure S2 and Table S4).

For comparability with previous work, we averaged exposure by trimester and entire pregnancy period. Associations with VTPB and MTPB were generally positive, but varied by trimester (see Supplemental Material, Table S5). We observed no evidence of effect measure modification for race/ethnicity, parity, smoking status, infant sex, state or region of birth, or by census-tract population density (data not shown). Inclusion of smoking as a covariate did not alter effect estimates (data not shown). Results of sensitivity analyses examining adjusting for temperature and season of conception were similar (see Supplemental Material, Table S6).

## Discussion

We examined associations between exposure to PM_2.5_ at each week of pregnancy and multiple categories of PTB. PM_2.5_ exposure in early and late gestational weeks and near-birth was associated with multiple categories of PTB, supporting the potential for multiple or overlapping pathways of action for PM_2.5_, based both on timing of exposure and severity of outcome.

Our results for PM_2.5_ exposures in early pregnancy were consistent with six studies across geographic areas that reported positive ORs (Chang et al. 2012; Hansen et al. 2006; Huynh et al. 2006; Jalaludin et al. 2007; Lee et al. 2012; Ritz et al. 2007). Studies have also reported positive associations for exposures late in pregnancy or near birth (Chang et al. 2012; Gehring et al. 2011; Hansen et al. 2006; Wilhelm and Ritz 2005). Chang et al. (2012) also observed positive associations for exposure to PM_2.5_ in the second trimester.

Inverse or null ORs with early pregnancy PM_2.5_ exposure have also been reported. Jalaludin et al. (2007) reported inverse ORs for exposures occurring in the first trimester during summer months only. Gehring et al. (2011) found inverse associations with PM_2.5_ exposures in the first trimester and last month before birth that attenuated or reversed after adjustment for region. Wilhelm et al. (Wilhelm and Ritz 2005; Wilhelm et al. 2011) reported inverse ORs with single-pollutant models of PM_2.5_ for exposures during early pregnancy; however, in the later study of a population from a similar area, analysis using multipollutant models (including criteria air pollutants and traffic-related air toxics) produced positive ORs (Wilhelm et al. 2011). Shifts in associations with regional or multipollutant adjustment suggest the importance of considering composition of pollutants co-occurring with PM_2.5_. It is possible that residual confounding due to co-pollutants is also a factor in our study; however, our estimates were robust to adjustment for co-occurring ozone.

Like ours, the study by Warren et al. (2012) evaluated weekly windows of exposure, reporting elevated RDs with PM_2.5_ exposure in weeks 4–22 of gestation. Although these results do not perfectly align with ours, possibly because of differences in PM_2.5_ composition in Texas compared with the northeast, the authors do corroborate our findings of positive associations with exposures in earlier weeks of gestation. Identification of increased associations in very specific vulnerable periods in development may aid in the elucidation of potential mechanisms.

Most studies assessed exposure using monitors or monitoring-based methods (e.g., kriging, land use regression). Although kriging and land use regression impute concentrations with complete spatial and temporal coverage, they are best suited for areas with reasonably dense spatial and temporal monitoring. Chang et al. (2012) examined PM_2.5_ exposure in North Carolina using both monitor data and monitor-corrected CMAQ data, reporting similar associations between PM_2.5_ and PTB for women with exposure information from both sources; but not all women had monitoring data. For our study, the use of monitor-corrected CMAQ data offered more complete spatial and temporal coverage, including regions for which monitor data was not available. This expands generalizability of our results over monitor-based studies restricted to urban centers. Our large population also allowed examination of EMM by several factors and detection of very small associations across multiple time windows of exposure.

In this work, we examined more specific definitions of PTB than < 37 weeks of gestation. The two other studies that used a more specific definition of PTB [27–36 weeks for Chang et al. (2012); 29–36 weeks for Darrow et al. (2009)] found opposing results to one another for the first trimester/month of pregnancy. But, they used different analytic methods: Chang et al. (2012) used time-to-event analysis, and Darrow et al. (2009) a time-series approach. Using a cohort study design, we found mostly positive RDs for these gestational ages and exposure windows, except for LPTBs. The etiology of PTB may differ greatly at different gestational weeks, as fetal development and periods of vulnerabilities shift rapidly. Using the four categories may have helped reveal differences in associations that would otherwise have been masked by collapsing all categories of PTB into a single outcome.

It is challenging to directly compare and interpret differences across studies because of different research methodologies (study designs, outcome definitions, and exposure assessments, metrics, and contrasts) (Stieb et al. 2012). In addition to the methodological differences noted above, differences in results across studies may also be explained by actual differences in PM_2.5_ composition over time and geographical area due to pollutant sources and meteorological conditions (Bell et al. 2007).

Like most studies of air pollution and PTB, we relied on imperfect exposure classification and the results may reflect residual or unmeasured confounding. Exposure misclassification may be attributable to the use of a model for exposure assessment (even with bias correction), the use of ambient rather than personal data, the use of a single residential point rather than a profile of where a woman’s time is spent, and the assumption that residence at birth was unchanged throughout pregnancy. These factors would likely be nondifferential by outcome, though not necessarily by confounding factors. Consequences of exposure misclassification on the observed associations are complicated to predict. True exposure may be higher or lower than assigned based on residence, depending on the pollution levels where a woman works, the amount of time she commutes, or the amount of time spent indoors, potentially biasing associations in either direction (Allen et al. 2012; Hodas et al. 2012). Results for exposures during individual weeks may also be confounded by exposures during temporally correlated weeks. Some bias may also be introduced through the women who were not able to be geolocated. There are demographic differences between these women and the included population, some of which may indicate a higher likelihood of exposure to worse air quality (Miranda et al. 2011). In addition, bias due to residual or unmeasured confounding may have arisen from the use of proxy variables for socioeconomic status (SES) or lack of data on important factors, such as maternal obesity. SES, though approximated in a manner similar to other studies, was not well defined and may not have fully captured the influence of SES on the PM_2.5_–PTB association. Area-level SES has been linked to PTB, and PM exposure may vary by neighborhood characteristics linked to SES; thus there may be some unmeasured confounding by area-level characteristics in our study. Unmeasured factors such as maternal obesity and diet may modify the association between PM_2.5_ and PTB, potentially masking associations in some subgroups. Again, such relationships are complex and many patterns are possible, potentially resulting in biases both toward and away from the null.

RDs as absolute measure of risk are easily interpretable and can be simply transformed into a number need to harm (NNH = 1/RD), providing information about how changes to exposure would be expected to affect public health. For example, with exposure during week 15 of gestation, NNHs for PM_2.5_ and LPTB correspond to 5,587, meaning that for every 1-μg/m^3^ increase in ambient PM_2.5_ concentrations for 5,587 pregnant women, 1 LPTB occurs (assuming associations are causal). For ExPTB the NNH is much higher, at 39,526; this is attributable to the rarity of ExPTB. Although magnitude of association for ExPTB may be small, this outcome also carries the most severe consequences and costs. Given the ubiquitous nature of exposure to PM_2.5_, even the small changes we observed may affect public health.

In general, the positive associations observed here and throughout the literature call for consideration of potentially complex and subtle mechanisms by which PM_2.5_ may play a role in PTB. Several pathways are possible. PM_2.5_ exposure has been associated with markers of systemic inflammation in humans that have been associated with preterm delivery, such as high-sensitivity C-reactive protein (CRP) and fibrinogen, and gestational hypertension (Lee et al. 2012, 2013). Maternal inflammation may alter placental vascular function (Backes et al. 2013). In a study of mice, Veras et al. (2008) found PM exposure altered placental morphology, decreasing maternal blood space volume and maternal–fetal surface ratio, and increasing fetal capillary proliferation with inhaled exposure to nonfiltered ambient air (mean PM_2.5_ = 27.5 μg/m^3^) versus filtered ambient air (mean PM_2.5_ = 6.5 μg/m^3^). Given the experimental findings in rodents and hypothesized mechanisms of preterm labor, it is plausible that these changes may lead to PTB through inadequate placental perfusion or impaired nutrient exchange (Bobak 2000; Kannan et al. 2006; Veras et al. 2012).

A variety of means have been proposed for the possible mechanisms of PM_2.5_ to act on PTB, including susceptibility to infection, disruption of placentation, and nutrient deprivation. PM_2.5_ has been shown to increase susceptibility to respiratory infections in toxicological studies, including influenza and respiratory syncytial virus in mice, potentially acting through impaired clearance of PM or pathogens (U.S. EPA 2009). Respiratory infections may either act as a primer for secondary infection (wherein immunological response to/clearance of secondary infection is reduced) or be an indicator of systemic inflammation. (Behrman and Butler 2007; U.S. EPA 2009; Wilhelm and Ritz 2005). Interruption of placental implantation, development, or function is another plausible mechanism, though at present only investigated nonhuman studies (Backes et al. 2013; Veras et al. 2008, 2012). Particulates may interfere in placental processes by the transfer of sorbed toxic compounds to the fetus or placenta, inflammatory processes including oxidative stress pathways, or by increasing susceptibility to infectious agents which in turn act on fetal development (Lee et al. 2012; U.S. EPA. 2009). Near-birth exposures may increase PTB risk by nutritional deprivation—for example, PM_2.5_ exposure may lead to inflammation leading to inadequate placental perfusion or a constricted umbilical cord, wherein the stressed fetus may produce pro-inflammatory cytokines which can trigger the cascade of events leading to labor and birth (Kannan et al. 2006). Some of these potential mechanisms would be exposure-timing restricted (e.g., disruption of placentation) whereas others may act throughout gestation (e.g., inflammation).

Characterizing exposure in two ways—anchored from LMP and lagged from birth—allowed us to examine different relationships between PM and PTB, hypothesizing two different potential mechanisms by which PM_2.5_ might act on PTB. Exposures anchored at gestational age allowed us to examine associations of PM_2.5_ exposure that may signal disruption in specific stages of pregnancy; associations identified with exposures anchored at birth may signal effects “triggering” early labor and birth. Examining these different hypotheses required different approaches. Our results indicate heterogeneity by both period of exposure and by stage of PTB should be considered in future analyses.

PM_2.5_ exposures in early and late gestation were associated with increased RD for PTB, although the specific windows of exposure associated with elevated RDs varied by PTB category. Our findings suggest that exposures beginning around the time of implantation and near birth may be of particular importance. Refined exposure windows and outcome definitions may have improved our ability to detect subtle associations. The ubiquitous nature of particulate matter means exposure increases the potential for harm, even when effect estimate magnitudes are small. Many properties of PM_2.5_ could be responsible for the observed associations, and further studies examining specific PM_2.5_ components or properties could add valuable information about the properties of particulate matter for which regulation or intervention should be targeted to reduce adverse outcomes.

## Supplemental Material

(589 KB) PDF

## References

[r1] Allen RW Adar SD Avol E Cohen M Curl CL Larson T 2012 Modeling the residential infiltration of outdoor PM_2.5_ in the Multi-Ethnic Study of Atherosclerosis and Air Pollution (MESA Air). Environ Health Perspect 120 824 830; 10.1289/ehp.1104447 22534026 PMC3385439

[r2] Backes CH, Nelin T, Gorr MW, Wold LE (2013). Early life exposure to air pollution: how bad is it?. Tox Lett.

[r3] Barnett AG 2011 Time-dependent exposures and the fixed-cohort bias [Letter] Environ Health Perspect 119 A422 A423; 10.1289/ehp.1103885 21968256 PMC3230453

[r4] Behrman RE, Butler AS, eds. (2007). Preterm Birth: Causes, Consequences, and Prevention.

[r5] Bell ML Dominici F Ebisu K Zeger SL Samet JM 2007 Spatial and temporal variation in PM_2.5_ chemical composition in the United States for health effects studies. Environ Health Perspect 115 989 995; 10.1289/ehp.9621 17637911 PMC1913582

[r6] Bobak M (2000). Outdoor air pollution, low birth weight, and prematurity.. Environ Health Perspect.

[r7] Brauer M Lencar C Tamburic L Koehoorn M Demers P Karr C 2008 A cohort study of traffic-related air pollution impacts on birth outcomes. Environ Health Perspect 116 680 686; 10.1289/ehp.10952 18470315 PMC2367679

[r8] Byun D, Schere KL (2006). Review of the governing equations, computational algorithms, and other components of the models-3 Community Multiscale Air Quality (CMAQ) modeling system.. Appl Mech Rev.

[r9] Chang HH, Reich BJ, Miranda ML (2012). Time-to-event analysis of fine particle air pollution and preterm birth: results from North Carolina, 2001–2005.. Am J Epidemiol.

[r10] Clean Air Act. (2012).

[r11] Darrow LA, Klein M, Flanders WD, Waller LA, Correa A, Marcus M (2009). Ambient air pollution and preterm birth: a time-series analysis.. Epidemiology.

[r12] Dominici F McDermott A Zeger SL Samet JM 2003 National maps of the effects of particulate matter on mortality: exploring geographical variation. Environ Health Perspect 111 39 44; 10.1289/ehp.5181 12515677 PMC1241304

[r13] Dominici F, Peng RD, Bell ML, Pham L, McDermott A, Zeger SL (2006). Fine particulate air pollution and hospital admission for cardiovascular and respiratory diseases.. JAMA.

[r14] Garcia VC, Gego E, Lin S, Pantea C, Rappazzo K, Wootten A (2011). An evaluation of transported pollution and respiratory-related hospital admissions in the state of New York.. Atmos Pollut Res.

[r15] Gehring U, Wijga AH, Fischer P, de Jongste JC, Kerkhof M, Koppelman GH (2011). Traffic-related air pollution, preterm birth and term birth weight in the PIAMA birth cohort study.. Environ Res.

[r16] Gilbert WM, Nesbitt TS, Danielsen B (2003). The cost of prematurity: quantification by gestational age and birth weight.. Obstet Gynecol.

[r17] Hansen C, Neller A, Williams G, Simpson R (2006). Maternal exposure to low levels of ambient air pollution and preterm birth in Brisbane, Australia.. BJOG.

[r18] Hodas N, Meng Q, Lunden MM, Rich DQ, Ozkaynak H, Baxter LK (2012). Variability in the fraction of ambient fine particulate matter found indoors and observed heterogeneity in health effect estimates.. J Expo Sci Environ Epidemiol.

[r19] Hogrefe C, Lynn B, Goldberg R, Rosenzweig C, Zalewsky E, Hao W (2009). A combined model-observation approach to estimate historic gridded fields of PM_2.5_ mass and species concentrations.. Atmos Environ.

[r20] Huynh M, Woodruff TJ, Parker JD, Schoendorf KC (2006). Relationships between air pollution and preterm birth in California.. Paediatr Perinat Epidemiol.

[r21] Jalaludin B Mannes T Morgan G Lincoln D Sheppeard V Corbett S 2007 Impact of ambient air pollution on gestational age is modified by season in Sydney, Australia. Environ Health 6 16; 10.1186/1476-069X-6-16 17553174 PMC1894960

[r22] Kannan S Misra DP Dvonch JT Krishnakumar A 2006 Exposures to airborne particulate matter and adverse perinatal outcomes: a biologically plausible mechanistic framework for exploring potential effect modification by nutrition. Environ Health Perspect 114 1636 1642; 10.1289/ehp.9081 17107846 PMC1665414

[r23] Knüppel S Stang A 2010 DAG program: Identifying minimal sufficient adjustment sets [Letter] Epidemiology 21 159 20010223 10.1097/EDE.0b013e3181c307ce

[r24] Lee PC, Roberts JM, Catov JM, Talbott EO, Ritz B (2013). First trimester exposure to ambient air pollution, pregnancy complications and adverse birth outcomes in Allegheny County, PA.. Matern Child Health J.

[r25] Lee PC, Talbott EO, Roberts JM, Catov JM, Bilonick RA, Stone RA (2012). Ambient air pollution exposure and blood pressure changes during pregnancy.. Environ Res.

[r26] Lewtas J (2007). Air pollution combustion emissions: Characterization of causative agents and mechanisms associated with cancer, reproductive, and cardiovascular effects.. Mutat Res.

[r27] Mathews TJ, MacDorman MF (2010). Infant mortality statistics from the 2006 period linked birth/infant death data set.. Natl Vital Stat Rep.

[r28] Miranda ML, Edwards SE, Keating MH, Paul CJ (2011). Making the environmental justice grade: the relative burden of air pollution exposure in the United States.. Int J Environ Res Public Health.

[r29] Olsson D Mogren I Forsberg B 2013 Air pollution exposure in early pregnancy and adverse pregnancy outcomes: a register-based cohort study. BMJ Open 3 e001955; 10.1136/bmjopen-2012-001955 PMC3585966 23386578

[r30] Ritz B, Wilhelm M, Hoggatt KJ, Ghosh JK (2007). Ambient air pollution and preterm birth in the environment and pregnancy outcomes study at the University of California, Los Angeles.. Am J Epidemiol.

[r31] Saigal S, Doyle LW (2008). An overview of mortality and sequelae of preterm birth from infancy to adulthood.. Lancet.

[r32] Sapkota A, Chelikowsky AP, Nachman KE, Cohen AJ, Ritz B (2012). Exposure to particulate matter and adverse birth outcomes: a comprehensive review and meta-analysis.. Air Qual Atmos Health.

[r33] Spiegelman D, Hertzmark E (2005). Easy SAS calculations for risk or prevalence ratios and differences.. Am J Epidemiol.

[r34] Stieb DM, Chen L, Eshoul M, Judek S (2012). Ambient air pollution, birth weight and preterm birth: a systematic review and meta-analysis.. Environ Res.

[r35] Strand LB Barnett AG Tong S 2011 Methodological challenges when estimating the effects of season and seasonal exposures on birth outcomes. BMC Med Res Methodol 11 49; 10.1186/1471-2288-11-49 21501523 PMC3102035

[r36] U.S. EPA (U.S. Environmental Protection Agency). (2009). Integrated Science Assessment for Particulate Matter (Final Report).. http://cfpub.epa.gov/ncea/cfm/recordisplay.cfm?deid=216546.

[r37] U.S. EPA (U.S. Environmental Protection Agency). (2012). National Ambient Air Quality Standards (NAAQS).. http://www.epa.gov/air/criteria.html.

[r38] U.S. EPA (U.S. Environmental Protection Agency). (2013a). Air Emissions Sources: Particulate Matter.. http://www.epa.gov/cgi-bin/broker?_service=data&_debug=0&_program=dataprog.national_1.sas&polchoice=PM.

[r39] U.S. EPA (U.S. Environmental Protection Agency). (2013b). National ambient air quality standards for particulate matter; final rule.. Fed Reg.

[r40] Veras MM, Damaceno-Rodrigues NR, Caldini EG, Maciel Ribeiro AA, Mayhew TM, Saldiva PH (2008). Particulate urban air pollution affects the functional morphology of mouse placenta.. Biol Reprod.

[r41] Veras MM, Guimarães-Silva RM, Caldini EG, Saldiva PH, Dolhnikoff M, Mayhew TM (2012). The effects of particulate ambient air pollution on the murine umbilical cord and its vessels: a quantitative morphological and immunohistochemical study.. Reprod Toxicol.

[r42] Warren J, Fuentes M, Herring A, Langlois P (2012). Spatial-temporal modeling of the association between air pollution exposure and preterm birth: identifying critical windows of exposure.. Biometrics.

[r43] Wilhelm M Ghosh JK Su J Cockburn M Jerrett M Ritz B 2011 Traffic-related air toxics and preterm birth: a population-based case-control study in Los Angeles County, California. Environ Health 10 89; 10.1186/1476-069X-10-89 21981989 PMC3204282

[r44] Wilhelm M Ritz B 2005 Local variations in CO and particulate air pollution and adverse birth outcomes in Los Angeles County, California, USA. Environ Health Perspect 113 1212 1221; 10.1289/ehp.7751 16140630 PMC1280404

[r45] World Health Organization. (2012). Preterm Birth. Fact Sheet no. 363.. http://www.who.int/mediacentre/factsheets/fs363/en/.

[r46] Wu J Ren C Delfino RJ Chung J Wilhelm M Ritz B 2009 Association between local traffic-generated air pollution and preeclampsia and preterm delivery in the South Coast Air Basin of California. Environ Health Perspect 117 1773 1779; 10.1289/ehp.0800334 20049131 PMC2801174

[r47] Wu J, Wilhelm M, Chung J, Ritz B (2011). Comparing exposure assessment methods for traffic-related air pollution in an adverse pregnancy outcome study.. Environ Res.

[r48] Zou G (2004). A modified poisson regression approach to prospective studies with binary data.. Am J Epidemiol.

