# Supplementary material for: Exposure to Fine Particulate Matter during Pregnancy and Risk of Preterm Birth among Women in New Jersey, Ohio, and Pennsylvania, 2000–2005
Source: Environ Health Perspect. 2014 May 30;122(9):992–7. doi: 10.1289/ehp.1307456 (PMC4154214; doi:10.1289/ehp.1307456)
Supplement: (589 KB) PDF [file ehp.1307456.s001.pdf]

Environmental Health Perspectives doi:10.1289/ehp.1307456

Supplemental Material

Exposure to Fine Particulate Matter during Pregnancy and Risk of Preterm Birth among Women in New Jersey, Ohio, and Pennsylvania, 2000–2005

Kristen M Rappazzo, Julie L Daniels, Lynne C Messer, Charles Poole, and Danelle T Lobdell

Table of Contents **Page**

Table S1. Further maternal and fetal characteristics across term and preterm birth categories for included pregnancies to women living in OH, PA, or NJ 2000-2005 **2**

Figure S1. Results for $PM_{2.5}$, exposures anchored at estimated LMP, with and without adjustment for ozone **3**

Figure S2. Results for $PM_{2.5}$, lagged exposures, with and without adjustment for ozone **5**

Table S2. Results [risk difference (95% confidence interval) per 1,000,000 pregnancies] for 1 µg/m^3 increase in $PM_{2.5}$, exposures anchored at estimated LMP, adjusted for ozone **7**

Table S3. Results [risk difference (95% confidence interval) per 1,000,000 pregnancies] for 1 µg/m^3 increase in $PM_{2.5}$, exposures anchored at estimated LMP, not adjusted for ozone **8**

Table S4. Results [risk difference (95% confidence interval) per 1,000,000 pregnancies] for 1 µg/m^3 increase in $PM_{2.5}$, exposures lagged before birth, as shown in Figure S2. **9**

Table S5. Results [risk difference (95% confidence interval) per 1,000,000 pregnancies] for 1 µg/m^3 increase in $PM_{2.5}$, large windows of exposure **10**

Table S6. Sampling of results [risk difference (95% confidence interval) per 1,000,000 pregnancies] for 1 µg/m^3 increase in $PM_{2.5}$ with adjustment for temperature, season, and ozone for VPTB and LPTB outcomes with exposures at week 5 or 15 of pregnancy **11**

Table S1. Further maternal and fetal characteristics across term and preterm birth categories for included pregnancies to women living in OH, PA, or NJ 2000-2005.

	ExPTB	VPTB	MPTB	LPTB	Term births
Observations (n)	8,664	12,004	31,446	90,037	1,639,376
**Infant sex**					
Male	4,536 (52)	6,368 (53)	16,781 (53)	48,017 (53)	835,429 (51)
Female	4,124 (48)	5,636 (47)	14,664 (47)	42,019 (47)	803,931 (49)
Missing	4 (<1)	0 (0)	1 (<1)	1 (<1)	16 (<1)
**Smoker**					
No	6,639 (77)	9,216 (77)	24,446 (78)	72,374 (80)	1,385,050 (84)
Yes	1,893 (22)	2,669 (22)	6,722 (21)	17,028 (19)	245,218 (15)
Missing	132 (2)	119 (1)	278 (1)	635 (1)	9,108 (1)
**Season of conception**					
Summer	1,977 (23)	2,846 (24)	7,300 (23)	21,054 (23)	387,660 (24)
Fall	2,360 (27)	3,304 (28)	8,763 (28)	24,869 (28)	459,926 (28)
Winter	2,357 (27)	3,224 (27)	8,436 (27)	24,165 (27)	439,416 (27)
Spring	1,970 (23)	2,630 (22)	6,947 (22)	19,949 (22)	352,374 (21)
**Parity**					
Primiparous	4,343 (50)	5,716 (48)	14,275 (45)	37,605 (42)	655,183 (40)
Multiparous	4,271 (49)	6,225 (52)	17,034 (54)	52,156 (58)	979,929 (60)
Missing	50 (1)	63 (1)	137 (<1)	276 (<1)	4,264 (<1)

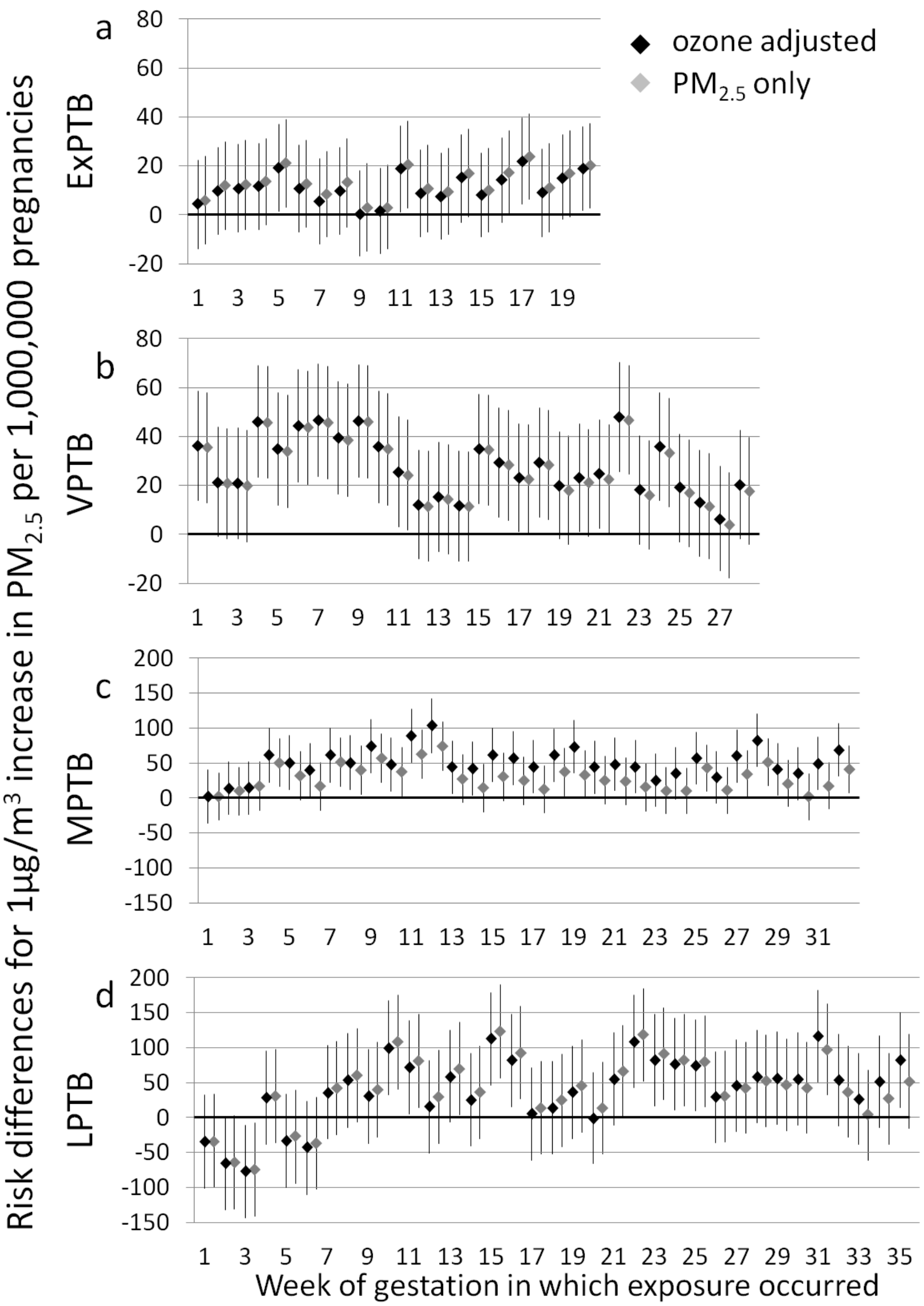

Figure S1. Results for $PM_{2.5}$, exposures anchored at estimated LMP, with and without adjustment for ozone. Risk differences for preterm birth with 1µg/m3 increases in particulate matter under 2.5 micrometers in aerodynamic diameter ($PM_{2.5}$) per 1,000,000 pregnancies for women residing in OH, PA, or NJ with pregnancies at risk of preterm birth from Jan 1, 2000 to Dec 31, 2005. Adjusted for maternal

race, education level, marital status, age at delivery (grey diamond) and co-occurring ozone (black diamond). Exposures are anchored at time of conception. a) outcome = risk of birth at 20-27 weeks of gestation (ExPTB), (b) outcome = risk of birth at 28-31 weeks of gestation (VPTB), (c) outcome = risk of birth at 32-34 weeks of gestation (MPTB), and (d) outcome = risk of birth at 35-36 weeks of gestation (LPTB). Numeric data are provided in Tables S2 and S3.

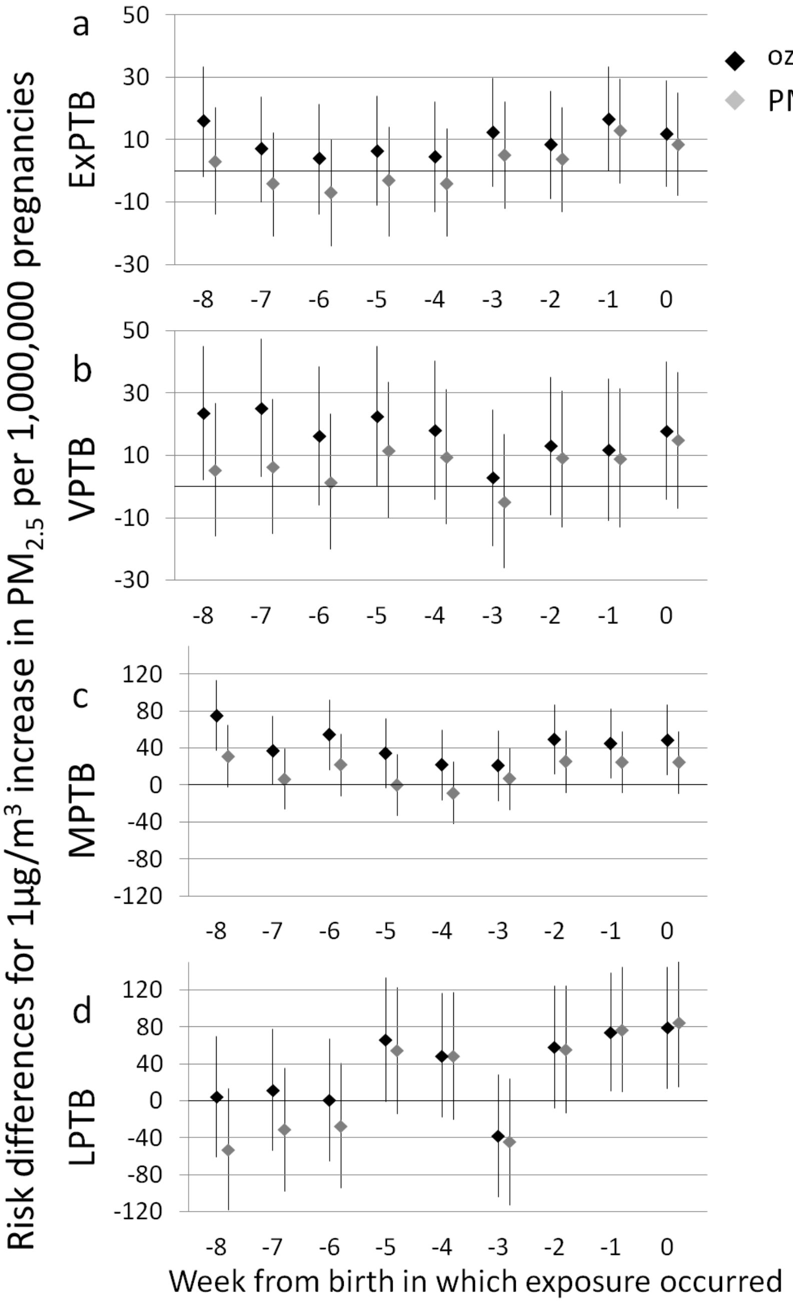

Figure S2. Results for $PM_{2.5}$, lagged exposures, with and without adjustment for ozone. Risk differences for preterm birth with 1 μg/m^3 increases in particulate matter under 2.5 micrometers in aerodynamic diameter ($PM_{2.5}$) per 1,000,000 pregnancies for women residing in OH, PA, or NJ with pregnancies at risk of preterm birth from Jan 1, 2000 to Dec 31, 2005. Adjusted for maternal race, education level, marital

status, age at delivery (grey diamond) and co-occurring ozone (black diamond). Exposures are anchored at birth. a) outcome = risk of birth at 20-27 weeks of gestation (ExPTB), (b) outcome = risk of birth at 28-31 weeks of gestation (VPTB), (c) outcome = risk of birth at 32-34 weeks of gestation (MPTB), and (d) outcome = risk of birth at 35-36 weeks of gestation (LPTB). Numeric data are provided in Table S4.

Table S2. Results [risk difference (95% confidence interval) per 1,000,000 pregnancies] for 1 μg/m^3 increase in $PM_{2.5}$, exposures anchored at estimated LMP, adjusted for ozone. As shown in Figures 1, S1, and S2.

Week of pregnancy in which exposure occurred	ExPTB	VPTB	MPTB	LPTB
1	4.4 (-14, 22.4)	36.2 (13.8, 58.6)	2.3 (-36, 40.2)	-35 (-101, 32.2)
2	9.8 (-8, 27.6)	21.2 (-1, 43.8)	13.9 (-24, 52)	-65 (-132, 1.7)
3	10.8 (-7, 28.8)	20.7 (-2, 43.5)	14.2 (-24, 52.2)	-77 (-144, -11)
4	11.8 (-6, 29.2)	46 (23.2, 68.9)	61.1 (22.6, 99.7)	28.5 (-39, 95.7)
5	19.2 (1.3, 37.1)	34.8 (11.8, 57.8)	50.5 (11.8, 89.3)	-33 (-100, 33.7)
6	10.7 (-7, 28.5)	44.4 (21.3, 67.4)	40.1 (1.8, 78.4)	-43 (-110, 23.1)
7	5.6 (-12, 23)	46.5 (23.5, 69.6)	61 (22.5, 99.5)	35.7 (-31, 102.7)
8	9.6 (-8, 27.4)	39.3 (16.3, 62.4)	50.4 (11.8, 89)	53.3 (-14, 120.4)
9	0.3 (-17, 18)	46.3 (23.3, 69.4)	74 (35.5, 112.5)	30.1 (-37, 97.4)
10	1.5 (-16, 18.9)	35.8 (13, 58.6)	47.8 (9.3, 86.3)	99.5 (32, 167.1)
11	18.8 (1.1, 36.4)	25.4 (2.9, 48)	88.8 (50.1, 127.4)	72.3 (5.5, 139.1)
12	8.9 (-9, 26.6)	12.1 (-10, 34.5)	103.5 (64.7, 142.4)	15.4 (-51, 81.9)
13	7.5 (-10, 25.1)	15.2 (-7, 37.5)	44 (6, 82)	58.6 (-7, 124.4)
14	15.2 (-3, 32.9)	11.6 (-11, 33.9)	41.9 (4.1, 79.8)	25.3 (-41, 91.4)
15	8.2 (-9, 25.3)	34.9 (12.4, 57.3)	61.4 (23.4, 99.3)	113.1 (46.5, 179)
16	14.3 (-3, 31.5)	29.3 (7, 51.7)	56.7 (18.7, 94.7)	81.7 (15.5, 147.8)
17	21.9 (4.4, 39.5)	23.2 (1.1, 45.3)	44.5 (7, 82.1)	5.2 (-61, 71.3)
18	9.1 (-9, 27)	29.4 (7, 51.7)	61.1 (23.3, 99)	13.8 (-52, 79.8)
19	15.1 (-2, 32.6)	19.7 (-2, 41.8)	73.3 (35.3, 111.3)	35.8 (-30, 101.8)
20	18.9 (1.6, 36.1)	23.1 (1.2, 45)	43.9 (6, 81.9)	-1 (-66, 64.7)
21		24.7 (2.4, 46.9)	48.1 (10.6, 85.6)	54.8 (-11, 121)
22		47.9 (25.7, 70.2)	44.5 (6.8, 82.3)	108.8 (42.5, 175)
23		18.1 (-4, 40.3)	25.3 (-12, 62.7)	81.9 (16, 147.8)
24		35.9 (13.7, 58)	35.1 (-2, 72.4)	76.1 (10.3, 142)
25		19.1 (-3, 40.8)	57.1 (19.8, 94.5)	74.3 (9.4, 139.2)
26		12.9 (-9, 34.5)	29.2 (-8, 66.6)	29.1 (-36, 94.4)
27		6.3 (-15, 27.9)	59.9 (22.2, 97.6)	45.3 (-20, 110.9)
28		20.3 (-2, 42.6)	82.5 (45, 120)	58.2 (-8, 124.5)
29			41 (3.9, 78.1)	55.9 (-10, 122.2)
30			34.7 (-3, 72.3)	55.2 (-11, 121.2)
31			49.4 (11.8, 86.9)	115.8 (50.2, 181.5)
32			68.6 (30.8, 106.4)	54 (-12, 119.7)
33				26.3 (-39, 92)
34				51.4 (-14, 116.9)
35				82.2 (14.1, 150.3)

Table S3. Results [risk difference (95% confidence interval) per 1,000,000 pregnancies] for 1 $\mu g/m^3$ increase in $PM_{2.5}$, exposures anchored at estimated LMP, not adjusted for ozone. As shown in Figures 1, S1, and S2.

Week of pregnancy in which exposure occurred	ExPTB	VPTB	MPTB	LPTB
1	5.8 (-12, 23.9)	35.4 (13, 57.8)	1.7 (-32, 35.8)	-34 (-100, 33.1)
2	11.9 (-6, 29.8)	20.7 (-2, 43.3)	9.8 (-25, 44.2)	-64 (-131, 2.3)
3	12.5 (-6, 30.6)	19.8 (-3, 42.6)	16.8 (-18, 51.3)	-74 (-141, -8)
4	13.6 (-4, 31)	45.8 (22.9, 68.7)	50.6 (15.9, 85.2)	30.7 (-36, 97.8)
5	21.1 (3.1, 39.1)	33.8 (10.8, 56.8)	32.1 (-3, 67)	-27 (-94, 39.2)
6	12.7 (-5, 30.6)	43.6 (20.5, 66.7)	16.5 (-18, 50.8)	-37 (-103, 29.4)
7	8.5 (-9, 26)	45.6 (22.5, 68.7)	51.7 (16.9, 86.5)	41.6 (-25, 108.5)
8	13.2 (-5, 31.2)	38.4 (15.4, 61.5)	39.6 (4.8, 74.4)	60.1 (-7, 127.2)
9	3 (-15, 20.9)	45.9 (22.8, 69)	57 (22.3, 91.7)	39.8 (-28, 107.2)
10	3 (-14, 20.4)	34.8 (12, 57.7)	37.3 (2.4, 72.1)	107.9 (40.4, 175.5)
11	20.5 (2.8, 38.2)	24.2 (1.6, 46.8)	62.3 (27.5, 97.2)	81.1 (14.3, 148)
12	10.6 (-7, 28.4)	11.5 (-11, 34)	73.9 (38.8, 108.9)	29.5 (-37, 96)
13	9.5 (-8, 27.3)	14.4 (-8, 36.7)	27.7 (-7, 62)	70 (4.1, 135.8)
14	17 (-1, 34.9)	11.3 (-11, 33.6)	14.1 (-20, 48.1)	36.3 (-30, 102.5)
15	10 (-7, 27.2)	34.5 (12, 57)	30.6 (-4, 64.8)	123.2 (56.6, 189.8)
16	17.2 (0, 34.5)	28.2 (5.7, 50.6)	24.4 (-10, 58.7)	92.5 (26.4, 158.6)
17	23.8 (6.2, 41.4)	22.6 (0.5, 44.7)	12.3 (-21, 45.8)	13.8 (-52, 79.8)
18	11.2 (-7, 29.2)	28.3 (6, 50.7)	37.1 (3, 71.2)	24.4 (-42, 90.4)
19	16.9 (-1, 34.5)	17.9 (-4, 40.1)	33 (-1, 67.2)	45.1 (-21, 111)
20	20.1 (2.8, 37.4)	21.1 (-1, 43)	25.4 (-9, 59.5)	13.4 (-52, 78.9)
21		22.5 (0.3, 44.8)	23.8 (-10, 57.4)	65.8 (0, 131.9)
22		46.7 (24.5, 68.9)	15.3 (-19, 49.2)	118.2 (52, 184.3)
23		16.1 (-6, 38.4)	10.1 (-23, 43.7)	91.4 (25.6, 157.2)
24		33.4 (11.3, 55.6)	9.8 (-23, 43.1)	81.7 (16, 147.4)
25		17 (-5, 38.6)	42.7 (9.3, 76.1)	80.2 (15.5, 144.9)
26		11.4 (-10, 33)	10.8 (-23, 44.2)	30.4 (-35, 95.4)
27		3.9 (-18, 25.4)	33.8 (0.2, 67.4)	42.6 (-22, 107.7)
28		17.6 (-4, 39.7)	50.9 (17.4, 84.5)	52.5 (-13, 118.2)
29			20.6 (-12, 53.6)	47 (-19, 112.7)
30			1.6 (-32, 35.1)	41.9 (-23, 107.3)
31			17.2 (-16, 50.7)	97.4 (32.5, 162.3)
32			41 (7.6, 74.4)	36.9 (-28, 101.8)
33				4 (-61, 68)
34				27 (-38, 91.6)
35				51.7 (-16, 118.9)

Table S4. Results [risk difference (95% confidence interval) per 1,000,000 pregnancies] for 1 $\mu g/m^3$ increase in $PM_{2.5}$, exposures lagged before birth, as shown in Figure S2.

Exposure week before birth	ExPTB	VPTB	MPTB	LPTB
**Adjusted for ozone**				
0	11.8 (-5, 28.9)	17.9 (-4, 40.1)	48.6 (10.8, 86.4)	78.9 (13.6, 144.2)
-1	16.6 (0, 33.4)	11.9 (-11, 34.5)	44.8 (7.5, 82.1)	74.3 (10.2, 138.3)
-2	8.5 (-9, 25.5)	13.1 (-9, 35)	49.4 (11.9, 86.9)	58.1 (-8, 123.8)
-3	12.3 (-5, 29.6)	2.9 (-19, 24.7)	21.1 (-17, 58.9)	-38 (-104, 28.2)
-4	4.4 (-13, 22.2)	18.2 (-4, 40.4)	21.7 (-16, 59.5)	48.8 (-18, 115.9)
-5	6.3 (-11, 24)	22.6 (0.3, 44.9)	34.3 (-3, 71.8)	65.8 (-1, 132.7)
-6	3.9 (-14, 21.3)	16.2 (-6, 38.4)	54.4 (16.5, 92.3)	1 (-65, 67)
-7	7 (-10, 23.7)	25.2 (3.2, 47.2)	37.4 (0.1, 74.7)	11.7 (-54, 77.5)
-8	15.9 (-2, 33.4)	23.6 (2.2, 45.1)	75.2 (37.6, 112.8)	4.1 (-61, 69.3)
**Not adjusted for ozone**				
0	8.4 (-8, 25.1)	14.9 (-7, 36.7)	24.6 (-9, 57.9)	74.8 (10.4, 139.2)
-1	12.9 (-4, 29.4)	9 (-13, 31.3)	25 (-8, 58)	67 (3.7, 130.3)
-2	3.6 (-13, 20.2)	9.1 (-13, 30.7)	25.2 (-8, 58.5)	47.7 (-17, 112.4)
-3	5.1 (-12, 22.2)	-5 (-26, 16.8)	6.7 (-27, 40.4)	-48 (-113, 17.8)
-4	-4 (-21, 13.5)	9.4 (-12, 31.2)	-9 (-42, 24.9)	39.5 (-26, 105.5)
-5	-3 (-21, 14)	11.5 (-10, 33.5)	0 (-33, 32.9)	47 (-19, 112.9)
-6	-7 (-24, 10.2)	1.4 (-20, 23.3)	21.8 (-12, 55.4)	-23 (-88, 42.3)
-7	-4 (-21, 12.1)	6.4 (-15, 28.1)	6.5 (-26, 39.5)	-17 (-82, 47.8)
-8	2.9 (-14, 20.2)	5.4 (-16, 26.6)	31.1 (-2, 64.4)	-32 (-96, 32.7)

Table S5. Results [risk difference (95% confidence interval) per 1,000,000 pregnancies] for 1 $\mu g/m^3$ increase in $PM_{2.5}$, large windows of exposure.

Exposure period	ExPTB	VPTB	MPTB	LPTB
Pregnancy	37.6 (-12, 87.2)	92.1 (33.1, 151)	97.1 (12.6, 181.6)	140.3 (-21, 301.1)
Trimester 1	26.6 (-4, 56.8)	93.2 (54.2, 132.2)	104.2 (45.3, 163.2)	36 (-78, 149.8)
Trimester 2[a]	34.9 (3.5, 66.3)	71.2 (32.6, 109.9)	71.7 (12.5, 130.9)	162.2 (47.2, 277.3)
Trimester 3[a]		29.3 (-20, 78.8)	63.1 (1.3, 125)	119.9 (2.7, 237.1)
Last month	23 (-1, 46.9)	22.1 (-9, 53.1)	50.6 (3.6, 97.5)	69.9 (-22, 161.4)

[a]Partial period for gestations not covering full trimester length.

Table S6. Sampling of results [risk difference (95% confidence interval) per 1,000,000 pregnancies] for 1 μg/m^3 increase in $PM_{2.5}$ with adjustment for temperature, season, and ozone for VPTB and LPTB outcomes with exposures at week 5 or 15 of pregnancy.

Adjusted for:	VPTB: week 5	VPTB: week 15	LPTB: week 5	LPTB: week 15
none	33.8 (10.8, 56.8)	34.5 (12, 57)	-27 (-94, 39.2)	123.2 (56.6, 189.8)
ozone (results shown)	34.8 (11.8, 57.8)	34.9 (12.4, 57.3)	-33 (-100, 33.7)	113.1 (46.5, 179)
temperature	33.0 (9.3, 56.6)	34.8 (11.9, 57.7)	-32 (-100, 36.4)	90.3 (22.3, 158.2)
season	33.7 (10.3, 57.2)	38.8 (15.4, 62.1)	-28 (-96, 39.9)	92.8 (23.2, 162.4)
ozone + temperature	29.9 (6.0, 53.8)	33.7 (10.6, 56.8)	-15 (-83, 54.2)	100.7 (32.0, 169.4)
ozone + season	32.3 (8.6, 55.9)	39.1 (15.5, 62.7)	-24 (-92, 44.0)	102.5 (32.4, 172.6)
temperature + season	32.8 (9.1, 56.6)	37.5 (14.0, 61.1)	-21 (-90, 47.2)	82.6 (12.5, 152.8)
ozone + temperature + season	29.3 (5.2, 53.4)	37.1 (13.3, 61.0)	-12 (-81, 57.2)	94.2 (23.0, 165.5)

All models are adjusted for maternal race, age at delivery, education level, and marital status.
